# Isolation and characterisation of novel lytic bacteriophages for therapeutic applications in biofilm-associated prosthetic joint infections

**DOI:** 10.1093/sumbio/qvae028

**Published:** 2024-10-28

**Authors:** Nathan J Burton, Luís D R Melo, Michaël F D Tadesse, Bethany Pearce, Evangelos Vryonis, Antonia P Sagona

**Affiliations:** School of Life Sciences, University of Warwick, Coventry CV4 7AL, United Kingdom; CEB—Centre of Biological Engineering, University of Minho, Campus de Gualtar, 4710-057 Braga, Portugal; LABBELS—Associate Laboratory, 4710-057 Braga, Portugal; School of Life Sciences, University of Warwick, Coventry CV4 7AL, United Kingdom; School of Life Sciences, University of Warwick, Coventry CV4 7AL, United Kingdom; Department of Infectious Diseases, University Hospital Coventry, Coventry CV2 2DX, United Kingdom; School of Life Sciences, University of Warwick, Coventry CV4 7AL, United Kingdom

**Keywords:** biofilms, phage therapy, bacteriophages, *Staphylococcus*, prosthetic joint infection, sequencing, antibiotic resistance, cytotoxicity

## Abstract

Prosthetic joint infections are devastating complications of joint arthroplasties. Without effective management, they can lead to limb amputation and even death. A significant proportion of these infections is caused by the primarily commensal Coagulase-negative *Staphylococci* pathogens, which form thick, antibiotic-resistant biofilms at the site of infection. Combinatorial therapy involving antibiotics and bacteriophages may represent a strategy to overcome resistance. Previous research indicates that as bacteria develop resistance to antibiotics, they often become more susceptible to bacteriophages. In this study, we produced a cocktail of novel bacteriophages and assessed their viability to eradicate nosocomial staphylococcal biofilms. Here, we used clinical isolates from prosthetic joint infections to isolate and identify four new bacteriophages from sewage effluent. These novel phages were characterized through electron microscopy and full genome sequencing. Subsequently, we combined them into a phage cocktail, which effectively re-sensitized biofilms to vancomycin and flucloxacillin. Notably, this phage cocktail demonstrated low cytotoxicity *in vitro* to human epithelial cells, even when used alongside antibiotic treatments. These findings highlight the potential of the phage cocktail as a tool to increase antibiotic treatment success in prosthetic joint infections.

**Sustainability Statement**

The research presented in this paper contributes to several UN Sustainable Development Goals (SDG’s):

1. UN SDG 2: Zero Hunger (Food Security)

While this paper does not directly address food security, the mitigation of infections and related complications contributes to overall well-being. By enhancing healthcare outcomes, this research supports food security by preserving physical health of individuals, allowing them to lead productive lives.

1. UN SDG 3: Good Health and Well-being (One Health)

This paper directly addresses SDG 3 by improving the health and well-being of individuals through the development of an adjuvant to antibiotics to improve patient outcomes.

1. UN SDG 14: Life Below Water (Ocean Sustainability)

Although this paper does not have a direct link to SDG 14, the demonstration of isolating a novel bacteriophage therapy from sewage effluent sheds light on the sort of ecology that is being discharged into from sewage treatment plants into our oceans.

1. UN SDG 13: Climate Action (Climate Action)

Through the proposal of phage therapy to extend the life of the antibiotics currently available, this reduces the costly need for more research and development of new small-molecule antibiotics.

1. UN SDG 1: End Poverty (Economic Equality)

This research demonstrated the relative ease of how bacteriophages can be isolated, propagated, and applied. The isolation of new bacteriophages has much lower cost overheads compared to small-molecule antibiotics. This research will hopefully influence policy to allow phage therapies in many countries and contribute to making them available to poor communities with phage therapies being low-cost, cutting-edge therapies.

## Introduction

Orthopaedic operations carry a significant risk of multidrug-resistant (MDR) infections, resulting in extremely high morbidity among patients, often leading to outcomes such as limb amputation and sepsis (Tande and Patel 2014). Nearly half of all prosthetic joint infections (PJIs) are caused by organisms within the Gram-positive *Staphylococcus* group (Tai et al. 2022)—*S. epidermidis, S. succinis, S. aureus, S. warneri*, and *S. capitis* are routinely found on the skin as commensal bacteria, contributing to the majority of infections (Joglekar et al. 2023). These bacteria can easily cross over from skin to surgical devices such as catheters, leading to severe biofilm-associated infections (Pihl et al. 2013, Becker et al. 2014). Given the abundance and strong antimicrobial resistance of the *Staphylococcus* genera, research on new therapeutics targeting *Staphylococcus* group of pathogens is a high-priority agenda for the World Health Organization (WHO) (Campoccia et al. 2006).

The widespread use of antibiotics has created selective pressures, leading to the emergence of resistant strains and limiting treatment options for many infections. Pathogens often employ biofilm formation to increase their resistance to antibiotics. Notably, *S. aureus* can form biofilms on orthopaedic devices, significantly contributing to the strong resistance profiles of infections affecting prosthetic joints. These biofilms consist of aggregated bacteria within an extracellular matrix (ECM). This matrix is a complex and highly polar mixture of biomolecules, including proteins, polysaccharides, nucleic acids, and lipids, all acting as a barrier, limiting ionic interactions and the diffusion of antibiotics (Farber et al. 1990, Overhage et al. 2008, Balcázar et al. 2015). Even when exposed to high antibiotic concentrations, individual surviving bacteria deep within biofilms can persist and re-establish more resistant biofilms (Ciofu et al. 2017). Treatment approaches involve invasive methods, such as debridement and replacement of implants contaminated with biofilms (Izakovicova et al. 2019).

The treatment of staphylococcal PJIs commonly involves the intravenous administration of antibiotics such as vancomycin, flucloxacillin, and rifampicin (Dan Córdoba et al. 2020). However, clinicians still regularly encounter infections resistant to these antibiotics (Sultan et al. 2022). One proposed solution is to use a combination of bacteriophages alongside antibiotic therapy as an adjunctive agent (Rahman et al. 2011). Bacteriophages, otherwise known as phages, are viruses that infect bacteria. The lytic life cycle of bacteriophages is defined by swift lysis of the host after infection, releasing progeny virions that go on to infect anew. Lytic bacteriophages have been previously demonstrated to exhibit strong bactericidal and anti-biofilm activity (Tkhilaishvili et al. 2018). Because of this, bacteriophages have been demonstrated to have the capability as an adjunctive agent alongside antibiotics to disrupt biofilms and significantly increase sensitivity of *Staphylococcus* biofilms to antibiotic therapy (Wang et al. 2020).

In this study, we describe the rapid development of a novel broad-range phage cocktail capable of re-sensitizing biofilms to antibiotics. We isolate and characterize four novel bacteriophages targeting a range of staphylococcal clinical infections, including the first bacteriophage isolated using *S. succinis*. The full genomes of these novel phages—ɸCF1, ɸCF5, ɸCF7, and ɸCF9—were determined using a combination of long-read and short-read sequencing. By combining these four bacteriophages, ɸCF1, ɸCF5, ɸCF7, and ɸCF9, with phage K (O’Flaherty et al. 2005), we created a phage cocktail with a wide host range across the *Staphylococcus* genus. Our study demonstrates the adjuvant effect of this phage cocktail in re-sensitizing biofilms to the antibiotic’s vancomycin and flucloxacillin, but not rifampicin. We achieved this by treating 15 biofilms of clinical isolates, sourced from PJIs, with a staggered treatment of phages followed by antibiotics. Furthermore, we evaluated the *in vitro* safety of using both phage cocktail and antibiotics to treat human epithelial cells.

## Materials and methods

### Bacterial strains and growth conditions

Fourteen clinical isolates of *Staphylococcus* (Supplementary Table S1) isolated from patients with infections post orthopaedic surgeries were provided by University Hospital Coventry pathology services. *Staphylococcus aureus* NCTC 9318 was obtained from the American Type Culture Collection (ATCC) and used as the reference strain for our studies and the propagation of phage K. *Staphylococcus* strains were cultured in Brain Heart Infusion (BHI) media and incubated at 37°C overnight.

### Bacteriophage enrichment and isolation from sewage effluent

Untreated sewage effluent was sampled from Finham sewage treatment works (Coventry, UK) and samples were promptly centrifuged at 1000 × *g* for 10 min. Resulting supernatant was filtered through a 0.22 µm syringe filter and incubated in a 1:1 ratio with cultures of *Staphylococcus* isolates in exponential phase in 2× LB medium, supplemented with 2 mM MgCl_2_/CaCl_2_, and left to incubate at 37°C overnight shaking at 180 rpm. After incubation, the samples were centrifuged at 8000 × *g* for 10 min and the resulting supernatant filtered to remove any residual bacteria. Double layer agar technique (DLA) (Stachurska et al. 2021) was performed on resulting phage enrichments to identify phage plaques, with resulting plaques being isolated and re-enriched in their host *Staphylococcus* isolate three times.

### Bacteriophage propagation

Bacteriophage was routinely propagated in Lysogeny broth (LB) broth supplemented with 2 mM MgCl_2_/CaCl_2_. A volume of 250 ml of supplemented LB broth was inoculated with an overnight culture of the complementary host organism to each bacteriophage strain and incubated at 37°C with shaking at 180 rpm. Once the culture reached an OD_600 nm_ of 0.1, phage was added to infect at a multiplicity of infection of 1. Infected cultures were incubated until lysis was observed (3–6 h), at which point cultures were centrifuged at 4000 × *g* for 20 min and the resulting supernatant filtered (pore size 0.22 µm) to remove any residual bacteria.

### Caesium chloride purification of bacteriophages

All phages used in this study were propagated as described above. For cytotoxicity studies with mammalian cell lines, bacteriophage was purified from bacterial endotoxins. To do this, NaCl was added to phage propagations to a final concentration of 1 M to precipitate bacterial proteins. After incubation on ice overnight, samples were centrifuged at 3220 × *g* and the supernatant passed through a 0.22-μm-pore-size membrane filter. PEG8000 was then added to a final concentration of 10% w/v and left overnight on ice to precipitate bacteriophage out of solution. Samples were centrifuged at 25 000 × *g* for 60 min and the resulting phage pellets resuspended in 6–7 ml SM buffer I (1 M NaCl, 8 mM MgSO_4_·7H_2_O, 25 mM Tris–HCl, pH 7.5) before being passed through a 0.2 μm-pore-size membrane filter. CsCl concentration and purification were performed in a density gradient CsCl solution for 20 h at 150 000 × g, 4°C. The extracted phage band was then dialysed first in SM buffer I and then with SM buffer II (100 mM NaCl, 8 mM MgSO_4_·7H_2_O, 25 mM Tris–HCl, pH 7.5). The resulting concentrated and purified bacteriophage samples were used in human cell assays.

### Electron microscopy

Phage isolate solutions were prepared to achieve titres of ∼1 × 10^8^ PFU/ml. Each sample was negatively stained with 1% uranyl acetate and electron micrographs were taken at various magnifications with a JEOL 2100Plus transmission electron microscope. ImageJ was used for annotating micrographs and measuring dimensions of bacteriophages.

### Bacteriophage host range

All cultures were incubated overnight at 37°C in BHI medium. A volume of 100 μl of each of the bacterial cultures were used to inoculate 0.5% BHI molten soft agar supplemented with 2 mM MgSO_4_/CaCl_2_ and overlaid on to BHI hard agar plates. Subsequently, 10 µl of each phage isolate (1 × 10^8^ PFU/ml) was spotted onto the prepared plates and incubated at 37°C overnight to obtain plaques. Host range was determined based on the clarity of the areas spotted with the bacteriophage sample (Kutter 2009).

### Bacteriophage genomic DNA purification

Bacteriophage genomic DNA was isolated using phenol–chloroform extraction (Jakočiūnė and Moodley 2018). Prior to DNA extraction, phage lysates were treated with DNase I and RNase A at room temperature for 1 h to remove any bacterial DNA and RNA contamination. The treated phage lysate was lysed for 1 h at 55°C in a lysis buffer containing 0.5 M ethylenediaminetetraacetic acid (EDTA), 10 mg/ml proteinase K, and 1% sodium dodecyl sulphate.. Phenol–chloroform purification was performed three times to remove any protein contamination. DNA quantification was performed using nanodrop (García-Alegría et al. 2020).

### Full genome sequencing of novel bacteriophages and bioinformatics analysis

Purified genomic DNA of each novel bacteriophage isolate was sequenced through short-read Illumina (provided by MicrobesNG) and long-read Oxford Nanopore sequencing (Sereika et al. 2022). Oxford Nanopore sequencing was performed using a modified protocol on a MINion flow cell to reach at least 100× coverage of each novel bacteriophage genome.

Then, reads for quality control and bacterial *de novo* assembly were performed using the INNUca v4.01 pipeline (https://github.com/BUMMI/INNUca), which consists of several integrated modules for reads QA/QC, *de novo* assembly, and post-assembly optimization steps. The sequencing data were uploaded to the Galaxy web platform, and we used the public server at usegalaxy.org to analyse the data (Afgan et al. 2016). Genomes were further assembled with Unicycler v0.4.8 (Wick et al. 2017). Assembled genomes were scanned through MyRAST (Aziz et al. 2008) for coding regions and results were manually curated using Geneious 9.0.5.

BLASTX was further used to check absent CDSs in intergenic regions. tRNAs were detected using tRNAscan-SE (Schattner et al. 2005) and ARAGORN (Laslett and Canback 2004). CDSs were queried against protein sequences in BLASTP (Altschul et al. 1990), and InterPro (Jones et al. 2014) for homology search and domain composition. CDSs transmembrane domains were predicted using TMHMM (Käll and Sonnhammer 2002) and Phobius (Käll et al. 2007). Comparative analysis was performed with BLASTN and EMBOSS Stretcher (Myers and Miller 1988). Virulence Factor Database (Liu et al. 2022) was used to check novel phages for *Staphylococcus* bacterial virulence factors.

### Nucleotide sequence accession number

The complete genome sequences of ɸCF1, ɸCF5, ɸCF7, and ɸCF9 were deposited in the National Center for Biotechnology Information (NCBI) database under accession numbers PP034387, PP034390.1, PP034388.1, and PP034389.1, respectively.

### Microtiter plate phage host virulence scores

Novel phage isolates ɸCF1, ɸCF5, ɸCF7, and ɸCF9 were compared against phage K for their virulence against their hosts. Fresh overnight cultures were adjusted with Mueller–Hinton Broth (MHB) to OD_600 nm_ ∼0.1 to achieve a working concentration of ∼10^8^ CFU/ml. Phage lysates were adjusted to a concentration of 1 × 10^8^ PFU/ml. For each assay, 180 µl of adjusted bacterial inoculate in MHB was mixed with 20 µl of phage in sterile Falcon (Corning) 96-well transparent plates to achieve a final phage concentration of 1 × 10^7^ PFU/ml. The plates were incubated at 37°C shaking at 200 rpm in a FLUOstar Omega plate reader (BMG LABTECH, Offenburg, Germany). Cell culture turbidity was monitored by measuring OD_600_ at 5-min intervals for 20 h and growth curves were produced by plotting OD_600_ against time. All assays were performed with three biological replicates. Latent periods were calculated from growth curves with the latent period initiated by phage addition at an MOI of 1 and the end of the latent period being defined by the steep drop in culture turbidity corresponding to phage-induced bacterial lysis (Paddison et al. 1998).

To assess the overall virulence of each phage against their hosts, we followed the methodology outlined by Xie et al. (2018). In this approach, growth patterns observed in each assay were summarized into single numerical values by calculating the area under the curve from the resulting phage-PJI isolate growth curves. These numerical values were then used to calculate virulence scores.

### Evaluation of antibiofilm activity by Calgary device

The activity of single antibiotics and phage cocktail-antibiotic combinations against biofilms was determined by the Calgary biofilm device for biofilm growth and microbroth dilution plates (Nunc™ Immuno TSP, Thermofisher Scientific). Three different antimicrobial agents commonly used for treatment of PJIs were used in combination with our phage cocktail: vancomycin, flucloxacillin, and rifampicin. Concentrations used were 128, 16, and 1 µg/ml for vancomycin, flucloxacillin, and rifampicin, respectively (Moses et al. 2020, Dan Córdoba et al. 2020, Campbell et al. 2023).

### Growth of mature biofilms

Overnight cultures of all strains were suspended in MHB to a working inoculum of ∼10^5^ CFU/ml, of which 160 µl was added to each well of a 96-well Calgary biofilm device (Nunc™ Immuno TSP, ThermoFisher Scientific) and incubated for 72 h at 37°C with shaking at 100 rpm. Experiments were put inside a humidity chamber to limit evaporation. Media was replaced with fresh MHB every 24 h to develop mature biofilms exposed to shear stress.

### Minimum biofilm eradicatory concentration determination

Once biofilms on textured pegs (Nunc™ Immuno TSP, Thermofisher Scientific) reached maturity, pegs were rinsed three times in 180 µl phosphate buffered saline (PBS) to remove planktonic bacteria before being placed into a treatment plate containing either only MHB or MHB containing 1 × 10^7^ PFU/ml phage cocktail (made up of phage K, ɸCF1, ɸCF5, ɸCF7, and ɸCF9). Pegs were incubated statically in control media or media containing phage cocktail for 24 h at 37°C. To perform staggered treatment of phage and then antibiotic, pegs were transferred from the phage treatment plate to an antibiotic treatment plant containing serially vancomycin, flucloxacillin, or rifampicin and incubated statically at 37°C statically for another 24 h. Starting concentration for vancomycin, flucloxacillin, and rifampicin was 128, 16, and 1 µg/ml. To assess the minimum biofilm eradicatory concentration (MBEC) in µg/ml of antibiotics required to eradicate biofilms, antibiotic-treated biofilms (with or without phage cocktail pretreatment) were washed 3 times in PBS to remove any residual planktonic bacteria and stamped onto a BHI hard agar plate and left to incubate at 37°C overnight. MBEC was determined by minimum concentration of antibiotic required to cause complete killing of the biofilm.

### Cytotoxicity assay

T24 urinary bladder epithelial cells (ATCC® HTB-4TM) were used for this study. Cytotoxicity to human cells was measured using the Invitrogen^TM^ CyQUANT^TM^ LDH Cytotoxicity Assay Kit, according to the protocols supplied with the kit.

T24 urinary bladder epithelial cells were grown at 37°C, 5% CO_2_ in tissue culture flasks containing McCoy’s 5A medium (ThermoFisher Scientific) supplemented with 10% foetal calf serum, 100 µg/ml penicillin, and 100 μg/ml streptomycin. Cells were then trypsinised and resuspended in medium to a final concentration of 400 000 cells/ml. A volume of 100 µl of this suspension was then added to 96-well plates and incubated overnight at 37°C with 5% CO_2_. The following day, CsCl purified bacteriophage cocktail was diluted in McCoy’s 5A modified medium to a final concentration of 1 × 10^7^ PFU/ml to 100 µl of phage/antibiotics media suspensions were added to corresponding wells and then samples were incubated for 24 h at 37°C with 5% CO_2_. The next day, 50 μl of supernatant from each well was transferred to the wells of a 96-well plate in triplicate before adding 50 μl LDH assay test solution. Samples were mixed by tapping the side of the plate and then covered with foil to protect them from light during a 30-min incubation at room temperature. As a control, 200 μl lysis buffer supplied with the kit was added to relevant samples for 45 min prior to adding LDH test solution. Fifty microlitres of stop solution was added to all samples before measuring the optical density (OD) at 490 nm and 680 nm. Relative cytotoxicity to human cell lines was calculated through OD_490 nm_ minus OD_680 nm_. All tests were carried out in triplicate with three biological replicates. Pairwise comparisons were performed using *t*-test, with a *P*-value of <0.05 being used as the threshold for significance.

### Statistical analysis

All experiments were completed in biological triplicate. Phage–antibiotic MBEC assays list median antibiotic concentrations alongside total variation seen across three biological replicates. Dunnett’s test was used for pairwise comparisons between phage cocktail/antibiotic cytotoxicity results.

## Results

### Clinical isolates of PJIs are resistant to antimicrobials

Clinical isolates were obtained from patients with PJIs. These infections exhibited varying resistance profiles to prescribed antibiotics at Coventry University Hospital, as detailed in Supplementary Table S1. Staphylococcal clinical isolate strains were identified through gram stains and illumina sequencing, which are documented in Supplementary Table S2.

### Isolation and characterisation of four novel lytic phages targeting MDR isolates

Municipal wastewater was sampled from Coventry Finham Sewage Treatment Works. These wastewater samples were used to screen for lytic phages against clinical isolates obtained from patients with PJIs. During the phage screening process, we discovered six new phages across a range of PJI clinical isolates. However, due to difficulties in propagating two of the novel phages, we focused our investigation on phages that could be successfully propagated using standard phage propagation protocols in liquid media. Specifically, we focused our studies on four of the isolated phages: ɸCF1, ɸCF5, ɸCF7, and ɸCF9.

**Figure 1.**
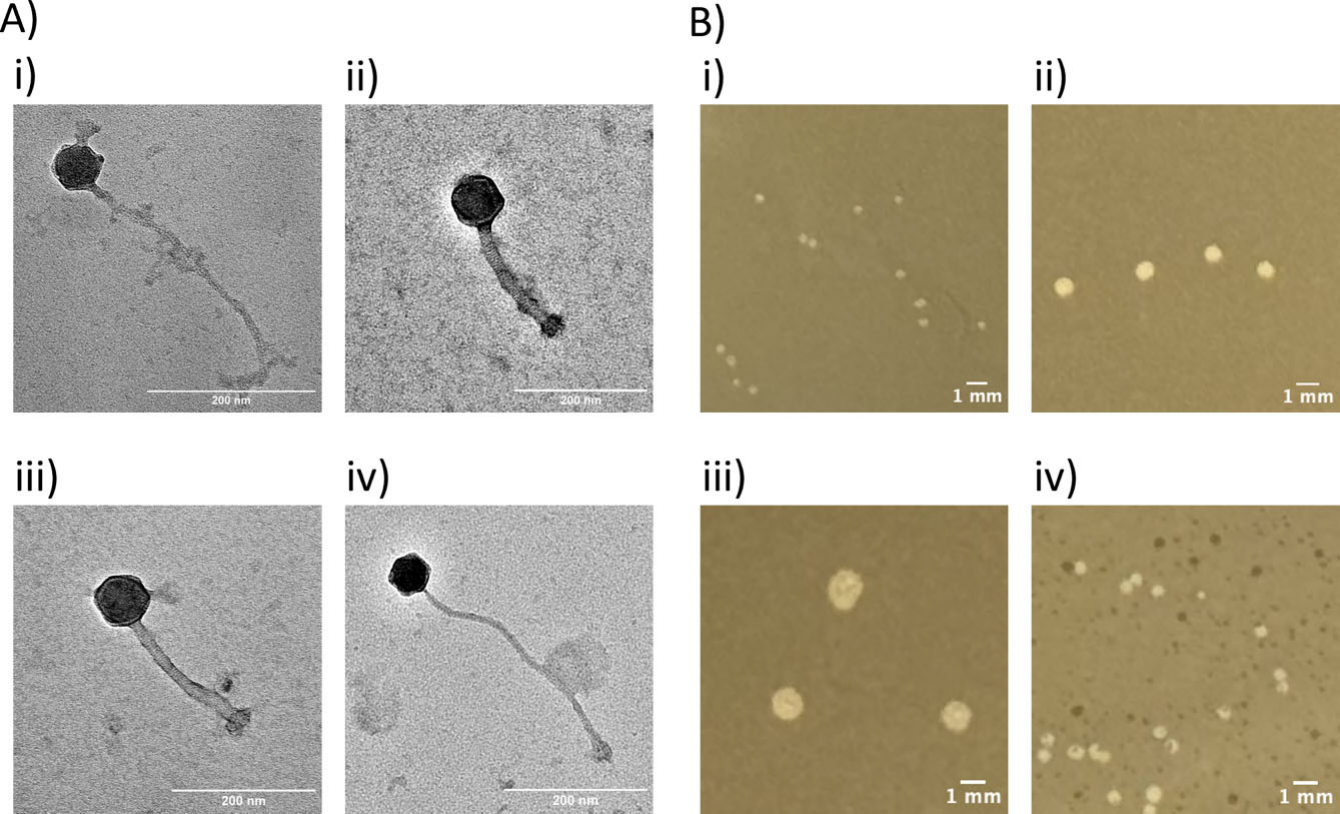
Staphylococcal phage isolates virion and plaque morphologies. (A) Transmission electron microscopy of the morphologies of novel phage isolates: (i) ɸCF1, (ii) ɸCF5, (iii) ɸCF7, (iv) ɸCF9. Scale bars represent 200 nm. (B) Morphology of individual phage plaques of (i) ɸCF1, (ii) ɸCF5, (iii) ɸCF7, (iv) ɸCF9. Scale bars represent 1 mm.

Transmission electron microscopy (TEM) data revealed the morphology of these phages (Fig. 1A). TEM images for the excluded phages are shown in Supplementary Fig. S1. The morphological features of ɸCF1 and ɸCF9 displayed long, non-contractile tails, while ɸCF5 and ɸCF7 exhibited contractile tails—the contracted states of ɸCF5 and ɸCF7 are depicted in Supplementary Fig. S2. Furthermore, we calculated the average morphological sizes of the novel phages, summarized in Supplementary Table S3.

In Fig. 1B, we observed that ɸCF1, ɸCF5, ɸCF7, and ɸCF9 all formed clear plaques, indicating lysis of the bacterial host cells. This finding suggests that all the phages we isolated exhibited lytic lifestyles.

### ɸCF1, ɸCF5, ɸCF7, and ɸCF9 presents expanded host range to PJIs

The host range of each phage against 15 different staphylococcal hosts was determined by spot testing and assessing the clearance of lawns of target bacteria after overnight incubation—clearance data for these isolates are presented in Supplementary Fig. S3. Overall clearance of bacterial lawns was categorized based on the relative clearance of bacterial lawns: no growth in the spotted area indicated sensitivity to the phage, some growth indicated medium sensitivity, and full growth indicated resistance. Table 1 summarizes the recorded sensitivities of each clinical isolate to individual phages and the phage cocktail. Notably, 7 out of 15 staphylococcal isolates were sensitive or showed at least medium sensitivity to our model phage K. However, when phage K was combined into a cocktail with ɸCF1, ɸCF5, ɸCF7, and ɸCF9, we successfully expanded the host range to 10 out of 15 tested staphylococcal isolates.

**Table 1.** Host range determination via spot testing phage formulations against PJI-associated clinical isolates.

Clinical Isolate	Species	Phage K	ɸCF1	ɸCF5	ɸCF7	ɸCF9	Cocktail
Reference	*Staphylococcus aureus* NCTC 9318	+	-	-	-	-	+
PJI1	*Staphylococcus epidermidis*	-	+	-	-	+	+
PJI2	*Staphylococcus epidermidis*	±	-	-	±	-	±
PJI3	*Staphylococcus aureus*	-	-	-	-	-	-
PJI4	*Staphylococcus epidermidis*	-	-	-	-	-	-
PJI5	*Staphylococcus warneri*	±	-	+	-	+	+
PJI6	*Staphylococcus aureus*	-	-	-	-	-	-
PJI7	*Staphylococcus succinus*	+	+	+	+	+	+
PJI8	*Staphylococcus aureus*	+	-	-	-	-	+
PJI9	*Staphylococcus epidermidis*	-	-	-	-	+	+
PJI11	*Staphylococcus epidermidis*	-	-	-	-	-	-
PJI12	*Staphylococcus aureus*	±	-	-	-	-	-
PJI13	*Staphylococcus epidermidis*	-	+	-	+	±	+
PJI14	*Staphylococcus capitis*	-	+	-	±	-	+
PJI16	*Staphylococcus epidermidis*	±	-	-	-	-	±

Ten microlitres of each phage lysate solution and phage cocktail with a titer of 1 × 10^8^ PFU/ml were pipetted onto lawns of clinical isolates bacterium. (+) sensitive (clear plaque); (+/−): medium (turbid plaque); (−): insensitive (no plaque).

### Sequencing of novel phages demonstrates lytic phenotypes, and no virulence-associated genes

Each novel bacteriophage was sequenced using both long read Oxford Nanopore sequencing and short-read Illumina sequencing. The complete genome sequence of each bacteriophage was deposited in the NCBI database and the general genomic features of the four bacteriophages are presented in Supplementary Fig. S4. Genomic analysis of the four phages revealed all bacteriophage genomes are composed of double-stranded DNA molecules, and phages ɸCF1, ɸCF5, and ɸCF7 have genes coding for tRNAs (Supplementary Table S4).

**Figure 2.**
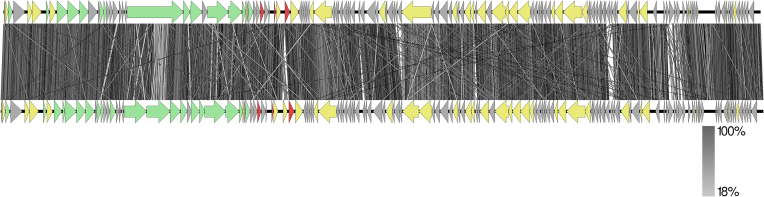
Sequence homology between the genomes of ɸCF1 (top) and ɸCF9 (bottom). ORFs are coloured according to their predicted functions: red for cell lysis, green for DNA packaging and structural proteins, yellow for DNA replication, recombination, and modification, and grey for hypothetical proteins. The genomes were compared using tbBLASTX within EasyFig (BLAST minimum length of 100 bp).

Three different subgroups of bacteriophages could be observed. The first one, composed of bacteriophages ɸCF1 and ɸCF9, presents similar genome sizes (∼93 kb), G + C content (∼29.3%), and a high nucleotide identity (92.2%). The sequence homology of this group is visualized in Fig. 2. The genomes in the first group share high nucleotide identity with other CoNS phages of the *Sextaecvirus* genus, namely, *Staphylococcus* phages vB_SepS_BE22, BESEP2, and 6ec, with identities above 97% and coverages above 80%. Consequently, we assigned these phages to the *Sextaecvirus* genus. The second group is composed by phage ɸCF5 presenting different sequence homology to most phages deposited on GenBank (<81%). ɸCF5 shares low nucleotide identity with other CoNS phages, namely *Staphylococcus* phages GH15, Stab20, and I30 with identities above 80% and coverages below 37%, being impossible to assign this phage to any current genus. The third group includes phage ɸCF7, which resembles other phages members of the *Twortvirinae* subfamily, namely *Staphylococcus* phages vB_Sau_S24, vB_Sau_Clo6, and Stab22, with identities above 95% and coverages above 92%. Although ɸCF7 can be assigned to the *Twortvirinae* subfamily, the International Committee on Taxonomy of Viruses (ICTV) did not create yet a genus to assign this phage, making it impossible to assign (Lefkowitz et al. 2018).

All the four phages were presumed lytic, with no coding sequences found for lysogenic life cycle markers (integrases, recombinases, and repressors). Furthermore, genomes were BLAST searched and compared against a virulence factor database by Liu et al. (2022), no virulence-associated or toxic coding sequences of significant coverage were matched (Supplementary Table S5), indicating that the isolated bacteriophages might be safe for therapy.

### Novel phages are more virulent against PJI-associated *Staphylococcus* infections than model phage

**Figure 3.**
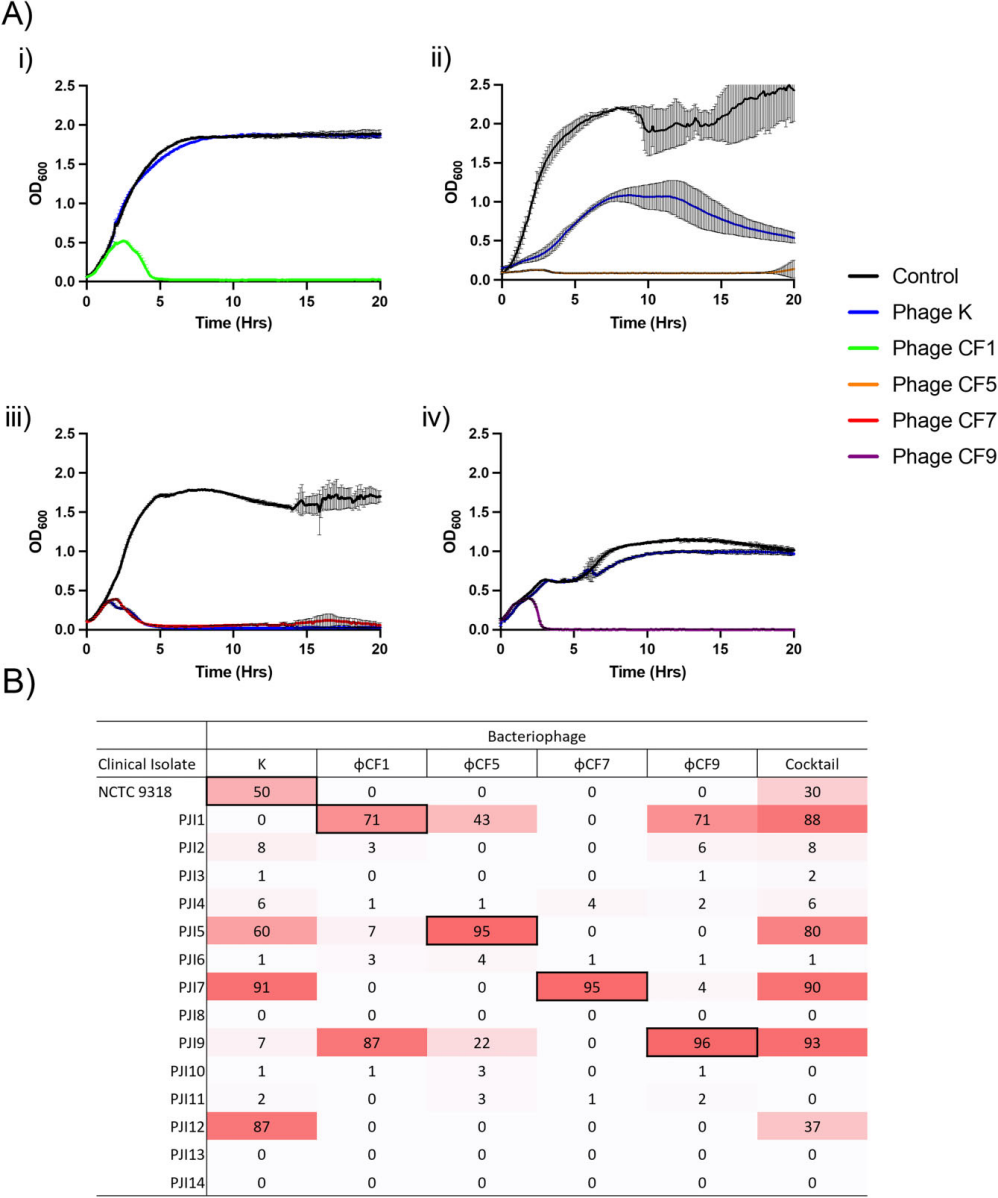
Virulence of novel phages. (A) Growth curves comparing the virulence of our novel phage isolates against PJI clinical isolates: (i) PJI1 (*S. epidermidis*), (ii) PJI5 *(S. warneri*), (iii) PJI7 (*S. succinis*), and (iv) PJI9 (*S. epidermidis*). (B) Virulence scores comparing overall virulence of each phage and phage cocktail to each PJI clinical isolate. Cells are colour-graded to assist the reader, with stronger colour intensity indicating a greater score. Larger numbers indicate greater suppression of bacterial growth in the presence of phage. Boxed cells indicate the initial isolation host of each novel phage. All experiments carried out at 1 × 10^7^ PFU/ml. Standard error for area under the curve for each phage/bacteria combination ranged from 0.01 to 1.15. Negative virulence values falling within one standard error were adjusted to zero.

We continued our investigation into the host range of the novel phages by performing growth curves with each of our novel phage isolates against planktonic clinical isolates, shown in Fig. 3A. The latent periods (time from initial infection to lysis of cultures) were estimated for the following phages: (i) ɸCF1—4 h, (ii) ɸCF5—3 h, (iii) ɸCF7—3.5 h, and (iv) ɸCF9—2 h. Over the 20-h period during which these growth curves were performed, resistance developed only to ɸCF5 at the 20-h mark.

To quantify the increased virulence of our novel bacteriophages compared to phage K, we used methods described by Xie et al. (2018) to produce growth curves (Supplementary Fig. S5) to create virulence scores of each phage against each clinical isolate in planktonic conditions. Virulence scores of each phage against each staphylococcal isolate are shown in Fig. 3B.

It was observed that all phages displayed a lower host range against our library of *Staphylococcus* strains in planktonic conditions, according to Xie et al.’s (2018) methods. Phage K displayed higher host range in spot tests, showing some kind of activity against 7/15 staphylococcal strains (Table 1), whereas in planktonic conditions, virulence scoring showed that phage K presented activity (score > 10.9) in only 4/15 of strains (Fig. 3B). Additionally, *S. succinis* (PJI7) was infected by all phages tested during spot testing (as shown in Supplementary Fig. S3). However, when we conducted virulence testing according to Xie et al.’s (2018) virulence scoring methodology, *S. succinis* only appeared to be susceptible to phage K and ɸCF7. Phage may either perform better when host is fixed in soft agar, or we may be observing ‘lysis from without’ (Abedon 2011).

ɸCF1, ɸCF5, ɸCF7, and ɸCF9 alongside phage K were combined into a phage cocktail and its virulence to planktonic cells were tested, shown in Fig. 3B. The phage cocktail presented significant virulence in 6/15 strains tested. Phage cocktail exhibited reduced virulence against PJI13 (*S. capitis*) compared to only phage K treatment. This reduction in virulence is likely due to competition with other phages within the cocktail, all vying for infection of *S. capitis*. Overall, different phages in phage cocktails appear to exhibit both agonistic and antagonistic effects toward each other.

### Novel bacteriophage cocktail can be used as an adjuvant to greatly increase the potency of antibiotics in eradicating biofilms

**Figure 4.**
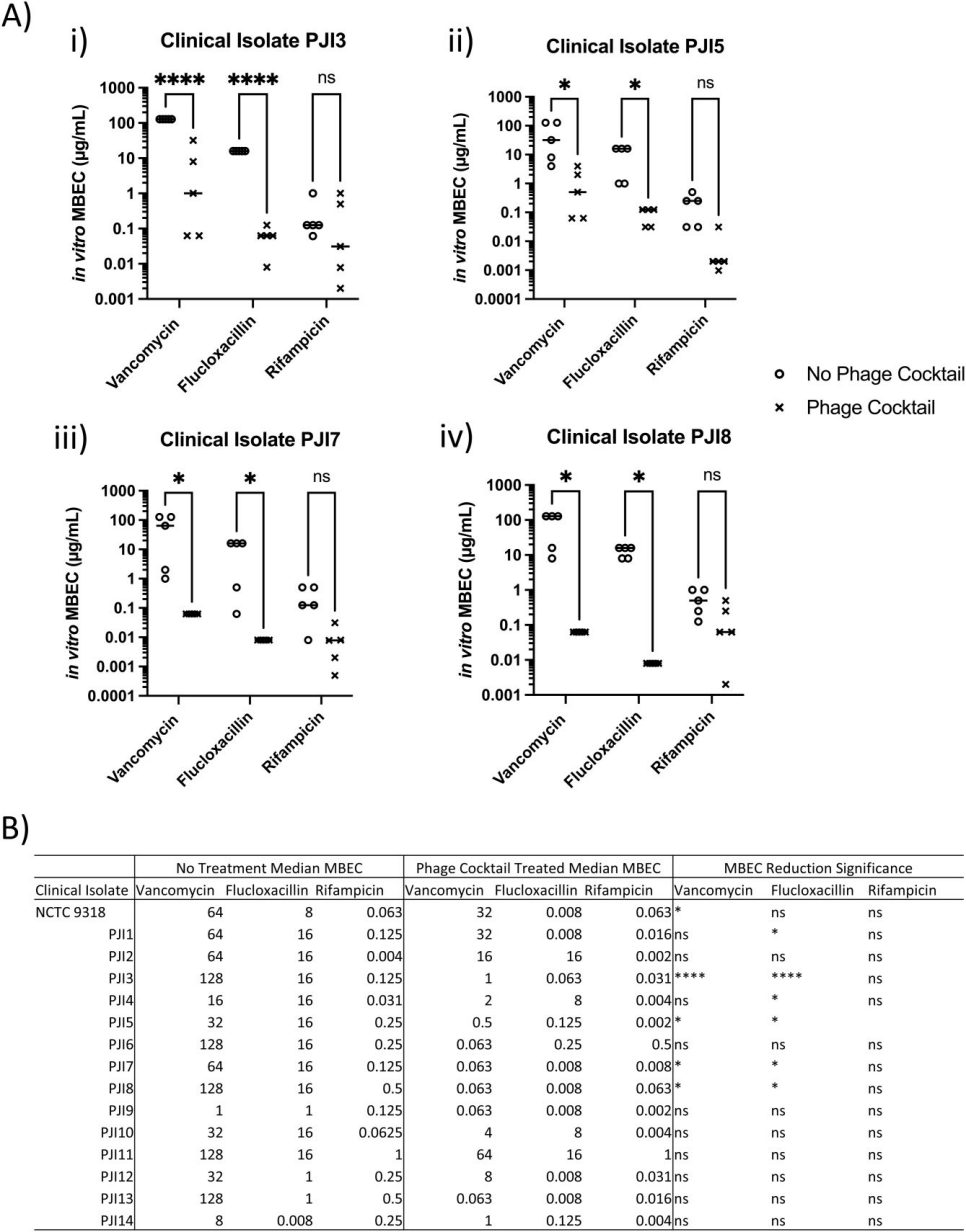
Phage cocktail increases the sensitivity of PJI biofilms to antibiotics. (A) Effect of phage cocktail on the MBEC (µg/ml) of vancomycin, flucloxacillin, and rifampicin against clinical isolates *in vitro*. Circles are MBEC values of biofilms treated without phage cocktail, and crosses represent MBEC values of phage cocktail treated biofilms (1 × 10^7^ PFU/ml). Bars in each graph indicate the median of five assays. (B) Table detailing median MBEC values of vancomycin, flucloxacillin, and rifampicin for PJI isolate biofilms treated with and without phage treatment. Data spread for each clinical isolate is shown in Supplementary Fig. S7. Significant MBEC reductions are denoted by *P < 0.0001, ****P < 0.05, and ns: no significance, by multiple Mann–Whitney tests with the Bonferroni–Dunn method, *n* = 5.

ɸCF1, ɸCF5, ɸCF7, and ɸCF9 alongside phage K were combined into a phage cocktail. The ability of this phage cocktail to reduce the concentration of antibiotics required to eradicate biofilms was evaluated by measuring the minimum concentration of antibiotics required to completely eradicate biofilms, known as the MBEC. Biofilms were grown by incubating *Staphylococcus* clinical isolates on textured pegs for 72 h with fresh media applied every 24 h. Mature biofilms on textured pegs were treated with a staggered treatment regime of phage cocktail and then antibiotics after 24 h as per previous findings by Wang et al. (2020). Wang et al. found that staggered treatment of biofilms by phage and then antibiotics was more effective than both treatments at the same time in reducing MBEC—we apply these findings to our methodology. MBEC values were defined as the lowest concentration of antibiotic (µg/ml) to yield no growth on the peg stamp plate (Supplementary Fig. S6). The adjuvant effect of this phage cocktail on the MBEC of each antibiotic is shown in Fig. 4. Figure 4A presents the most striking cases of biofilm re-sensitization to antibiotics, while Supplementary Fig. S8 shows the mucoid phenotype displayed by PJI7 (*S. succinis*) after treatment with antibiotics. All remaining MBEC values for phage cocktail vs. no phage cocktail biofilm eradication experiments are shown in Fig. 4B. The data spread of MBEC values for each antibiotic/phage combination against each clinical isolate is shown in Supplementary Fig. S7.

The phage cocktail significantly reduced the concentration of antibiotics required to eradicate biofilms when using vancomycin or flucloxacillin. However, the phage cocktail does not increase biofilm sensitivity to antibiotics in all cases: pretreatment of biofilms with the phage cocktail led to a significant decrease in MBEC in 5 out of 15 clinical isolates for vancomycin and 6 out of 15 clinical isolates for flucloxacillin. This finding demonstrates an adjuvant effect, where phage cocktail can weaken the resistance of biofilms to vancomycin and flucloxacillin.

Rifampicin exhibited a distinct behaviour; phage cocktail pretreatment did not cause any significant decrease in rifampicin’s MBEC across all clinical isolate biofilms tested. An explanation for this may be that the MBEC for rifampicin was already very low (≤0.25 µg/ml) across biofilms of all our clinical isolates without the phage cocktail pretreatment. This suggests that rifampicin already has high bioavailability within biofilms, leaving little room for improvement for phage cocktail.

Collectively, the phage cocktail presented adjuvant activity for antibiotics in 7/15 clinical isolates, suggesting potential for further activity with other antibiotics. When acting as an adjuvant to antibiotics for use against biofilms, the phage cocktail has a much broader host range of activity compared to the activity observed in our planktonic experiments in Fig. 3B. This suggests that this phage cocktail had effective phage–antibiotic antibiofilm activity even in non-host species.

### Phage cocktail presents no difference in overall cytotoxicity to human epithelial cells compared to antibiotics

**Figure 5.**
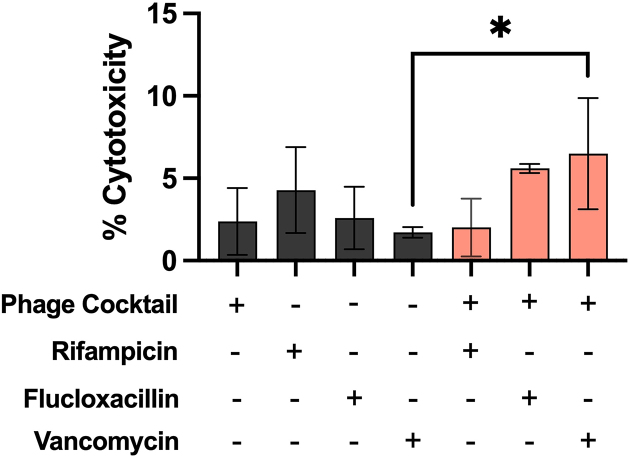
Cytotoxicity of novel phage cocktail to human epithelial cells. T24 human epithelial cells were incubated for 24 h with our phage cocktail (1 × 10^7^ PFU/ml) alongside vancomycin (128 µg/ml), rifampicin (1 µg/ml), and flucloxacillin (16 µg/ml). The cytotoxicity of phage–antibiotic combinations was also assessed. LDH cytotoxicity assays were performed using the Invitrogen CyQUANT kit. Error bars indicate standard error mean. Dunnett’s test was used for pairwise comparisons. *P ≤ 0.05, *n* = 3.

To assess the safety of our phage cocktail as a phage therapy to humans in an orthopaedic environment, the overall cytotoxicity of our phage cocktail was tested against T24 human bladder epithelial cells. T24 human bladder epithelial cells were treated with caesium chloride-purified phage cocktail alongside vancomycin, flucloxacillin, and rifampicin. We report no significant difference in overall cytotoxicity to human epithelial cells between purified phage cocktail at concentrations of 1 × 10^7^ PFU/ml and clinical concentrations of vancomycin, flucloxacillin, and rifampicin (Student’s *t* test, *P* > 0.05) (Fig. 5).

The cytotoxicity of purified phage cocktail when combined with antibiotic treatments was also tested against human epithelial cells. As shown in Fig. 5, T24 cells human epithelial cells displayed no difference in cytotoxicity when treated with the phage cocktail and flucloxacillin/rifampicin, indicating that phage cocktail can be safely used alongside these antimicrobials.

The T24 human epithelial cells exhibited a significant increase in cytotoxicity with co-treatment of vancomycin and our phage cocktail (Dunnett’s multiple comparisons test, *P* < 0.05). However, this is only a 4% increase in cytotoxicity compared to vancomycin alone. Considering this, some caution may be advised in using vancomycin alongside our novel phage cocktail to treat PJIs.

## Discussion

The formation of biofilms is a key factor in the resistance of implant-associated PJIs necessitating the use of powerful last-line antibiotics such as vancomycin, flucloxacillin, and rifampicin (Zheng and Stewart 2002, Zaborowska et al. 2017). With resistance to these last-line antibiotics becoming commonplace in PJIs, disrupting these biofilms must be a key target in increasing therapeutic success in chronic debilitating cases of PJIs (Singh et al. 2010, Reiter et al. 2012).

This study describes the isolation of four novel anti-staphylococcal phages targeting a range of clinical isolates of *Staphylococcus* originating from PJIs. These phages were isolated from samples collected from local sewage treatment plants. Previously, members of the *Kayvirus* genus, including phage K, have been reported as strong candidates for controlling *Staphylococcus-*associated infections. However, in our initial host range experiments, phage K appeared to exhibit lytic activity against only 7 out of 15 of our PJI isolates. Therefore, we sought to isolate new phages that can target a broader range of our *Staphylococcus* clinical isolates. Using sewage effluent sourced from Finham sewage treatment works, we discovered four novel bacteriophages targeting *Staphylococcus*-associated PJIs: ɸCF1, ɸCF5, ɸCF7, and ɸCF9. Notably, ɸCF7 appears to be the first phage in literature isolated with *S. succinis* as its host.

Before considering our novel phages for phage therapy, we verified that our bacteriophages preferred a lytic life cycle; phages that undergo lysogenic life cycles are not ideal for therapeutic use, due to the potential to render bacteria more virulent if the phage harbours deleterious genes (Suh et al. 2022). Our novel phages were confirmed to be lytic by checking for the absence of lysogeny markers. These markers can be physical, such a sturbid plaques, or genetic, such as the presence of integrases, recombinases, and repressors in the phage genomes, which allow them to integrate their genetic material into the host (Skurnik et al. 2007, Gill and Hyman 2010). The genomes of all four novel phages lacked lysogeny-associated genes. Additionally, the phage genomes showed no significant sequence homology with the Liu et al. (2022) Virulence Factor Database. Sequencing of our patient-specific phages found two new bacteriophages of a similar group, ɸCF1 and ɸCF9, having similar genetic identities to the already characterized *S. epidermidis* phage vB_SepS_BE22, suggesting they could be mutants of the same species of phage (Melo et al. 2014). ɸCF5 and ɸCF7 were found to be in completely new genera, with very low genetic identity to any other *Staphylococcus* phages.

We combined phages ɸCF1, ɸCF5, ɸCF7, and ɸCF9 with phage K to create a wide host range anti-staphylococcal phage cocktail. After pre-treating PJI-associated *Staphylococcus* biofilms with the phage cocktail, we observed an increased susceptibility of biofilms to vancomycin and flucloxacillin. Our clinical isolate biofilms were found to be sensitive to rifampicin; hence, we found no significant decrease in MBEC between biofilms treated without and with the phage cocktail. There may have been little scope for the phage to improve rifampicin’s bioavailability, as rifampicin is a potent biofilm eradicator (Dan Córdoba et al. 2020, Thill et al. 2022). Another reason for this observation may be that rifampicin’s mechanism of action is through the inhibition of bacterial DNA-dependent RNA polymerase—a process that may inhibit the phage’s ability to infect and propagate within the target bacterium (Calvori et al. 1965). This phenomenon was also observed by Wang et al. (2020), where phage Sb-1 had no adjuvant effect with rifampicin in treating *Staphylococcus* biofilms. Understanding this, believe our phage cocktail holds promise for significantly improving the success of vancomycin and flucloxacillin treatment in clinical settings.

For the screening phage host range, spot tests are the usual assay to use (Clokie 2009). However, the phage cocktails performance varies dramatically depending on the assay used. Our studies found that spot test results did not cross over with our liquid assay results. When performing spot test assays, high concentrations of phage may cause a ‘lysis from without effect’, as described by Abedon (2011), where the high concentration of phage and the relatively low concentration of bacteria in the soft agar may kill the bacteria without any infection. In infection assays using liquid culture, the presence of phage defence systems such as the viperin homologue MoaA in *S. aureus* can delay or prevent culture collapse (Fenwick et al. 2017, Bernheim et al. 2021). With any assays relating to biofilms, biofilms feature ‘persister’ cells with very low metabolic activity, which are more resistant to predation by phage and may re-colonize biofilms after phage predation (Verstraeten et al. 2016). The biofilm matrix can also contain ‘absorption traps’, which are decoy receptors to which phages bind, preemptively triggering the release of their DNA and preventing the phage reaching their target host cells (Pires et al. 2017). An example of this is the excess of teichoic acids in the ECM of biofilms, which mediate the adhesion of cells to the ECM protein fibronectin (Hussain et al. 2001). This highlights the importance of using different assays when assessing phage host range.

The safety of phage cocktail was assessed against human cells and was found to be no more cytotoxic to human cells than any standard antibiotic used for treating PJIs. Additionally, this phage cocktail can be used concurrently with antibiotics as an adjuvant to re-sensitize biofilms to antibiotics without increasing toxicity to human epithelial cells.

We did observe a small but significant increase in cytotoxicity when our phage cocktail was used in conjunction with vancomycin. Fujiwara et al. (2012) reported that vancomycin colocalizes in the endosomes of renal cells, activating cathepsins, promoting interferons, and triggering apoptosis. In a more recent study, Williams et al. (2023) found that phage K1F also co-localizes with endosomes. Taken together, when vancomycin and phage are used in combination, there may be an agonistic effect in promoting inflammation in epithelial cells.

It is worth noting that the concentrations of phages and vancomycin used in these experiments were high—10^7^ PFU/ml for the phage cocktail and 128 µg/ml for vancomycin, with therapeutic serum concentrations normally in the range of 15–20 µg/ml (Rybak et al. 2009). Research by other groups indicates that phage titres as low as 10^6^ PFU/ml effectively reduce the number of *S. aureus* in skin infections in murine models (Pincus et al. 2015, Shetru et al. 2021). Importantly, the levels of drug-induced cell death remained below those of certain therapeutics currently available. Previous studies using lactate dehydrogenase assays have demonstrated that phages are compatible with human epithelial cells. Styles et al. (2020) reported that vPhT2, a phage targeting *A. baumannii*, induced minimal cytotoxicity in human T24 bladder epithelial and brain vascular endothelial cells (hCMECs). Similarly, Kosznik-Kwaśnicka et al. (2023) found that two of their three staphylococcal phages resulted in a slight but statistically significant reduction in viability and metabolic activity of human skin fibroblasts.

In summary, here we report the isolation and characterisation of four novel lytic anti-staphylococcal phages. The phages presented potential as an adjunctive therapy for biofilm-associated PJIs. When the phages are combined into a cocktail, comprising of ɸCF1, ɸCF5, ɸCF7, and ɸCF9, they can re-sensitize antibiotic-resistant staphylococcal biofilms to vancomycin and flucloxacillin, significantly reducing the required antibiotic concentrations for biofilm eradication. Importantly, the phage cocktail exhibited low cytotoxicity *in vitro* to human epithelial cells, underscoring its safety for therapeutic applications. Together, these findings highlight the promise of phage therapy as a complementary approach to enhance antibiotic efficacy and combat antibiotic resistance in PJIs.

## Supplementary Material

qvae028_Supplemental_File

## Data Availability

The complete genome sequences of ɸCF1, ɸCF5, ɸCF7, and ɸCF9 were deposited in the National Center for Biotechnology Information (NCBI) database under accession numbers PP034387, PP034390.1, PP034388.1, and PP034389.1, respectively. The GenBank accession numbers of completed Illumina sequencing of all clinical isolates used in this study are shown in Supplementary Table S2.
