# Supplementary material for: Isolation and characterisation of novel lytic bacteriophages for therapeutic applications in biofilm-associated prosthetic joint infections
Source: Sustain Microbiol. 2024 Oct 28;1(1):qvae028. doi: 10.1093/sumbio/qvae028 (PMC13375367; doi:10.1093/sumbio/qvae028)
Supplement: Supplementary file 1 [file qvae028_Supplemental_File.docx]

# Supplementary Material Figures

| Clinical Isolate | Antibiotic Susceptibility | | | | | | | | |
| --- | --- | --- | --- | --- | --- | --- | --- | --- | --- |
|  | Ciprofloxacin | Cotrimoxazole | Flucloxacillin | Fusidic Acid | Linezolid | Rifampicin | Teicoplanin | Vancomycin | Clindamycin |
| PJI1 | I | S | S | R | S | S | S | S | S |
| PJI2 | R | R | R | S | S | S | S | S | R |
| PJI3 | I | S | S | S | S | S | S | S | R |
| PJI4 | R | R | R | R | S | S | S | S | R |
| PJI5 | I | S | S | S | S | S | S | S | - |
| PJI6 | R | S | S | S | S | S | S | S | R |
| PJI7 | I | S | S | R | S | S | R | S | S |
| PJI8 | I | S | S | S | S | S | S | S | R |
| PJI9 | R | R | R | R | S | S | S | S | S |
| PJI11 | R | R | S | S | S | S | S | S | S |
| PJI12 | R | S | S | S | S | S | S | S | S |
| PJI13 | I | S | S | R | S | S | S | S | S |
| PJI14 | I | S | S | R | S | - | S | S | S |
| PJI16 | I | S | S | R | S | S | S | S | S |

**Table 1: Host range determination via spot testing phage formulations against PJI-associated clinical isolates.** 10 µl of each phage lysate solution and phage cocktail with a titer of 1x10^8^ PFU/ml was pipetted onto lawns of clinical isolates bacterium. (+) sensitive (clear plaque); (+/−): medium (turbid plaque); (−): insensitive (no plaque).

| Clinical Isolate | Species | Genome Size (bp) | GC % | Accession number |
| --- | --- | --- | --- | --- |
| PJI1 | *Staphylococcus epidermidis* | 2461198 | 32.03 | JAZDAS000000000 |
| PJI2 | *Staphylococcus epidermidis* | 2602270 | 31.88 | JAZDAT000000000 |
| PJI3 | *Staphylococcus aureus* | 2690102 | 32.82 | JAZDAU000000000 |
| PJI4 | *Staphylococcus epidermidis* | 2537963 | 32.13 | JAZDAV000000000 |
| PJI5 | *Staphylococcus warneri* | 2500643 | 32.59 | JAZDAW000000000 |
| PJI6 | *Staphylococcus aureus* | 2769957 | 33.26 | JAZDAX000000000 |
| PJI7 | *Staphylococcus succinus* | 2829875 | 32.95 | JAZDAY000000000 |
| PJI8 | *Staphylococcus aureus* | 2799842 | 32.7 | JAZDAZ000000000 |
| PJI9 | *Staphylococcus epidermidis* | 2533325 | 31.85 | JAZDBA000000000 |
| PJI11 | *Staphylococcus epidermidis* | 2570085 | 32 | JAZDBB000000000 |
| PJI12 | *Staphylococcus aureus* | 2781702 | 32.69 | JAZDBC000000000 |
| PJI13 | *Staphylococcus capitis* | 2500423 | 32.72 | JAZDBD000000000 |
| PJI14 | *Staphylococcus capitis* | 2499600 | 32.71 | JAZDBE000000000 |
| PJI16 | *Staphylococcus epidermidis* | 2546159 | 31.91 | JAZDBF000000000 |

**Supplementary Table 2: GenBank accession numbers and genomic features from Illumina sequencing of PJI clinical isolates.**

**Supplementary Figure 1: A) *S. epidermidis* phage 1.1 and B) *S. aureus* phage 3.2.**

**Supplementary Figure 2: TEM images of contracted state of the contractile tail phages.** A) ɸCF5 and B) ɸCF7. Scale bar represents 200 nm.

| Phage | Head diameter | Tail length | Tail Width |
| --- | --- | --- | --- |
| ɸCF1 | 66 nm | 338 nm | 9 nm |
| ɸCF5 | 79 nm | 207 nm | 21 nm |
| ɸCF7 | 77 nm | 206 nm | 19 nm |
| ɸCF9 | 63 nm | 354 nm | 10 nm |

**Supplementary Table 3: Average dimensions of novel bacteriophages used in this study.**

**Supplementary Figure 3: Images illustrating 10 µL spot assays of 1x10^8^ PFU/mL phage lysate on each clinical isolate of phage K, ɸCF1, ɸCF5, ɸCF7, ɸCF9, and our phage cocktail of all 5 phages.**

ɸCF5

ɸCF7

ɸCF1

ɸCF9

**Supplementary Figure 4: Genome overview of novel phages characterised in this study.** Genome maps with the predicted CDSs (arrows) numbered and coloured according to their predicted function: blue for DNA replication and transcription genes, green for DNA packaging and phage morphogenesis genes, red for cells lysis genes, and yellow for hypothetical proteins. Some important CDSs are highlighted. The nucleotide position (in kb) is indicated above the genome. The figure was generated using Geneious 9.1.4. Sequence annotation detailed in methods.

| Phage | Accession Number | Genome Size (bp) | GC % | CDSs | tRNA | Closest Homolog | Family/Genus |
| --- | --- | --- | --- | --- | --- | --- | --- |
| ɸCF1 | PP034387 | 96,354 | 29.3 | 137 | tRNA-Pro-TGG | Staphylococcus phage vB_SepS_BE22 | Unclassified/ Sextaecvirus |
| ɸCF5 | PP034390.1 | 135,050 | 29.3 | 184 | tRNA-Asn-GTT | Staphylococcus phage GH15 | Unclassified/ unclassified |
| ɸCF7 | PP034388.1 | 138,217 | 31.0 | 204 | tRNA-Pseudo-TGG; tRNA-Asp-GTC | Staphylococcus phage vB_Sau_S24 | Herelleviridae/unclassified |
| ɸCF9 | PP034389.1 | 97,081 | 29.3 | 146 |  | Staphylococcus phage vB_SepS_BE22 | Unclassified/ Sextaecvirus |

**Supplementary Table 4: GenBank accession numbers and general genomic features from short and long-read genome sequencing of novel bacteriophages.** CDS = Coding Sequence.

A)

| **Sequence** | **Length (bp)** | **E-value** | **Overall Identity** | **Gaps** | **Query Aligned** | **Subject Aligned** |
| --- | --- | --- | --- | --- | --- | --- |
| VFG002406 (esaA) type VII secretion system protein EsaA [Type VII secretion system] [Staphylococcus aureus subsp. aureus MW2] | 3030 | 0.001 | 100.0% (22/22) | 0 | 34872-34893 | 1476-1497 |
| VFG001376 (cps4L) UDP-N-acetylglucosamine 2-epimerase [Capsule] [Streptococcus pneumoniae TIGR4] | 1185 | 0.005 | 100.0% (21/21) | 0 | 70175-70195 | 719-699 |
| VFG041267 (lem7) Dot/Icm type IV secretion system effector Lem7 [Dot/Icm] [Legionella pneumophila subsp. pneumophila str. Philadelphia 1] | 2310 | 0.005 | 90.9% (30/33) | 0 | 52870-52902 | 66-98 |
| VFG006567 (fliH) flagellar assembly protein H [Pse5Ac7Ac] [Helicobacter pylori 26695] | 777 | 0.005 | 96.0% (24/25) | 0 | 29653-29677 | 405-429 |
| VFG002294 (C2I) C2 toxin (component-I) [C2 toxin] [Clostridium botulinum C str. 203U28] | 1296 | 0.005 | 100.0% (21/21) | 0 | 66457-66477 | 780-800 |

B)

| **Sequence** | **Length (bp)** | **E-value** | **Overall Identity** | **Gaps** | **Query aligned** | **Subject aligned** |
| --- | --- | --- | --- | --- | --- | --- |
| VFG001795 (cba) c protein beta antigen [-C protein] [Streptococcus agalactiae FM027022] | 3252 | 0.008 | 100.0% (21/21) | 0 | 95761-95781 | 446-466 |
| VFG045529 (lpg1639) Dot/Icm type IV secretion system effector [Dot/Icm] [Legionella pneumophila subsp. pneumophila str. Philadelphia 1] | 1317 | 0.008 | 100.0% (21/21) | 0 | 53770-53790 | 131-111 |
| VFG002295 (C2II) C2 toxin (component-II) [C2 toxin] [Clostridium botulinum C str. 203U28] | 2166 | 0.008 | 100.0% (21/21) | 0 | 51560-51580 | 1903-1923 |

C)

| **Sequence** | **Length (bp)** | **E-value** | **Overall Identity** | **Gaps** | **Query aligned** | **Subject aligned** |
| --- | --- | --- | --- | --- | --- | --- |
| VFG001534 (efa1) EHEC factor for adherence [Efa-1/LifA] [Escherichia coli O111:H- str. E45035] | 9672 | 0.002 | 100.0% (22/22) | 0 | 144938-144959 | 1347-1326 |
| VFG001995 (flgF) flagellar basal body rod protein FlgF [Flagella] [Legionella pneumophila subsp. pneumophila str. Philadelphia 1] | 747 | 0.007 | 100.0% (21/21) | 0 | 59031-59051 | 578-598 |

D)

| **Sequence** | **Length (bp)** | **E-value** | **Overall Identity** | **Gaps** | **Query aligned** | **Subject aligned** |
| --- | --- | --- | --- | --- | --- | --- |
| VFG002406 (esaA) type VII secretion system protein EsaA [Type VII secretion system] [Staphylococcus aureus subsp. aureus MW2] | 3030 | 0.001 | 100.0% (22/22) | 0 | 35601-35622 | 1476-1497 |
| VFG001376 (cps4L) UDP-N-acetylglucosamine 2-epimerase [Capsule] [Streptococcus pneumoniae TIGR4] | 1185 | 0.005 | 100.0% (21/21) | 0 | 71838-71858 | 719-699 |
| VFG041267 (lem7) Dot/Icm type IV secretion system effector Lem7 [Dot/Icm] [Legionella pneumophila subsp. pneumophila str. Philadelphia 1] | 2310 | 0.005 | 90.9% (30/33) | 0 | 53596-53628 | 66-98 |
| VFG006567 (fliH) flagellar assembly protein H [Pse5Ac7Ac] [Helicobacter pylori 26695] | 777 | 0.005 | 96.0% (24/25) | 0 | 30380-30404 | 405-429 |
| VFG002294 (C2I) C2 toxin (component-I) [C2 toxin] [Clostridium botulinum C str. 203U28] | 1296 | 0.005 | 100.0% (21/21) | 0 | 68121-68141 | 780-800 |

**Supplementary Table 5: BLAST search alignment with Liu et al. (2022) core virulence factor database for phages A) ɸCF1, B) ɸCF5, C) ɸCF7, and D) ɸCF9.**

**Supplementary Figure 5: Growth curves of PJI clinical isolate library after infection with each novel phage/cocktail at a concentration of 1x10^7^ PFU/mL over a 10-hour period.** Blue = clinical isolate only, red = phage K, green = ɸCF1, purple = ɸCF5, orange = ɸCF7, black = ɸCF9, brown = phage cocktail.

**Supplementary Figure 6:** MBEC biofilm assays stamped onto square BHI agar plates with biofilms exposed to serially diluted concentrations of A) vancomycin (128 µg/mL), B) rifampicin (1 µg/mL), and C) flucloxacillin (16 µg/mL) with (i) or without (ii) phage cocktail treatment (1x10^6^ PFU/mL).

**Supplementary Figure 7: Effect of phage cocktail on the *in vitro* MBEC of vancomycin, flucloxacillin and rifampicin.** Circles indicate MBEC values of biofilms, and crosses indicate MBEC values of phage cocktail treated biofilms (1x10^7^ PFU/mL). Bars in each graph indicate the median of five assays. ****: p < 0.0001, *: p < 0.05 by Multiple Mann-Whitney tests with Bonferroni-Dunn method.

**Supplementary Figure 8: A) Non-mucoid and B) mucoid phenotypes of PJI7 after overnight incubation on BHI Agar plates at 37 ^o^C.** The mucoid phenotype regularly appears after refrigeration of plates.
